# Supplementary material for: Heat Shock Transcription Factor CgHSF1 Is Required for Melanin Biosynthesis, Appressorium Formation, and Pathogenicity in Colletotrichum gloeosporioides
Source: J Fungi (Basel). 2022 Feb 11;8(2):175. doi: 10.3390/jof8020175 (PMC8876323; doi:10.3390/jof8020175)
Supplement: Supplementary file 1 [file jof-08-00175-s001.zip › Supplementary Figures and Table S1.pdf]

Supplementary files

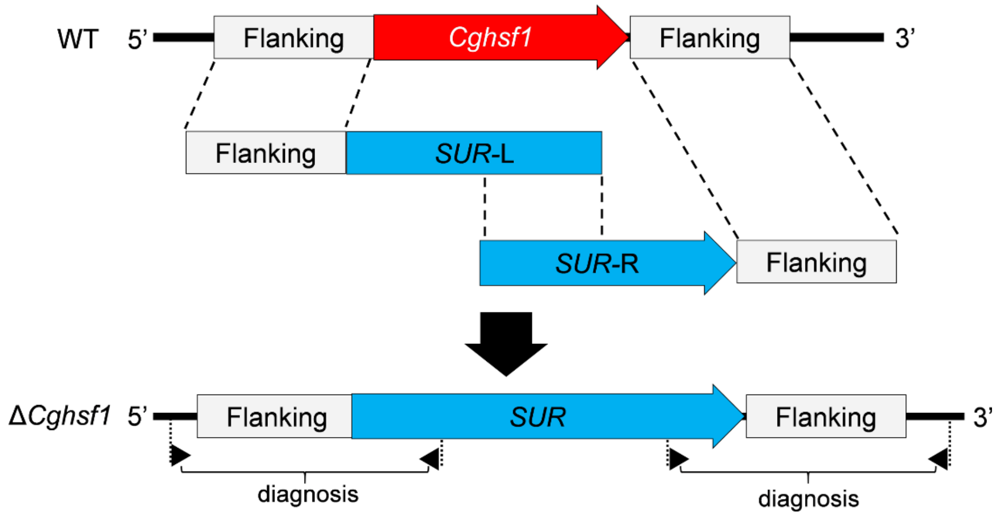

Figure S1. Split-Marker Strategy for construction of *Cghsf1* knock-out mutants. The acetolactate synthase gene (*SUR*) cassette from *Magnaporthe oryzae*, which confers resistance to chlorimuron ethyl, was used as selective marker. Diagnostic primers for integrations of the recombinant fragments are marked with black triangles. WT: wide type; ΔCgHSF1: the knock-out mutants.

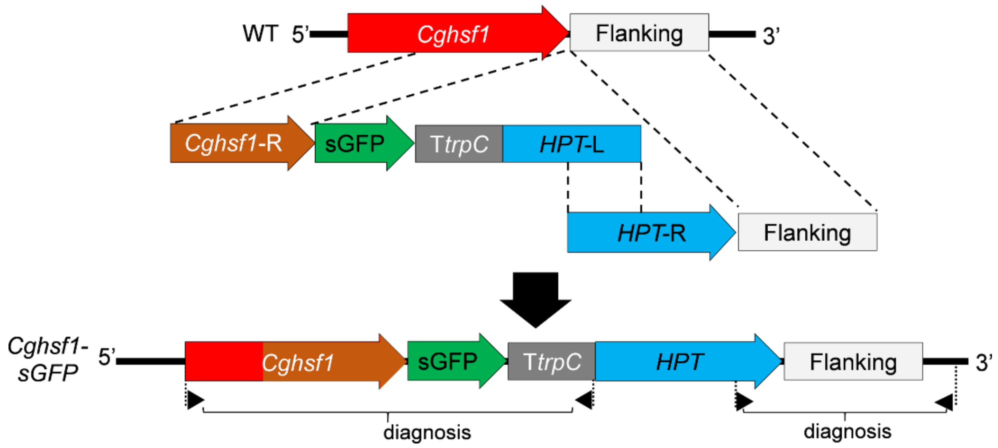

Figure S2. Strategy for construction of *CgHSF1-sGFP* mutant. The hygromycin B resistance cassette (*HPT*) was used as the selective marker. Diagnostic primers for integrations of the recombinant fragments are marked with black triangles. Cghsf1-R: The 700 bp nucleotides of 3′ part of *Cghsf1* without stop codon.

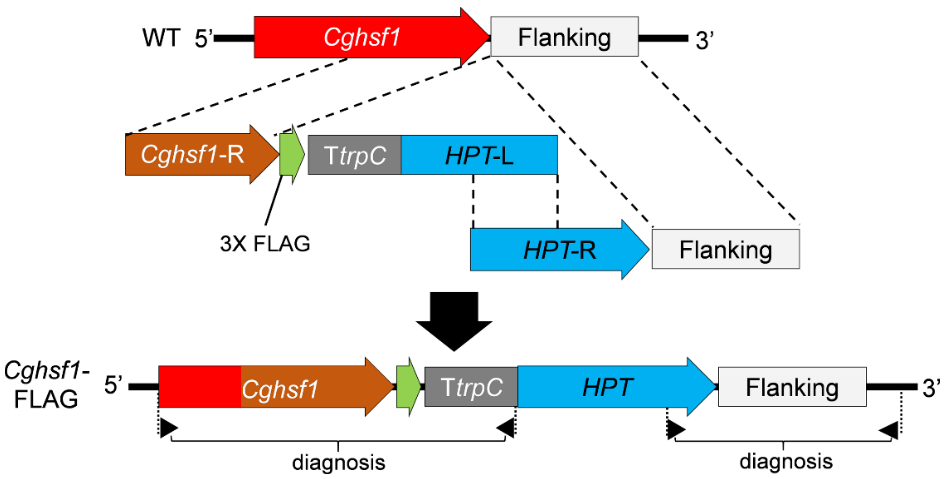

Figure S3. Strategy for construction of *CgHSF1*-FLAG mutant. The hygromycin B resistance cassette (*HPT*) was used as the selective marker. Diagnostic primers for integrations of the recombinant fragments are marked with black triangles. Cghsf1-R: The 700 bp nucleotides of 3′ part of *Cghsf1* without stop codon.

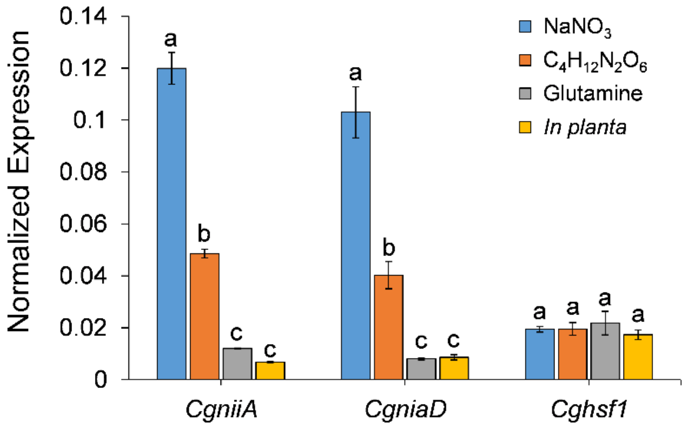

Figure S4. Relative expression levels of *CgniiA*, *CgniaD*, and *Cghsf1* of WT strain cultured *in vitro* on nitrogen sources of $NaNO_3$ (20 mmol L^{-1}), ammonium tartrate ($C_4H_{12}N_2O_6$) (10 mmol L^{-1}), and Glutamine (10 mmol L^{-1}) and during *in planta* stage. The β2-tubulin coding gene was used as an endogenous control for normalization. Columns with different letters indicate significant difference at $p < 0.05$.

Table S1. Nucleotide sequence of *Cghsf1* and the amino acid sequence of CgHSF1. The red letters indicate the intron.

**>*CgHSF1* Nucleotide sequence**
ATGCCGATGCCAGCAGTTCAGCAGTCTTTTCCCAGCCCGACGGGCGATCAGTTT
TTGCGATGGGGAGGAGCAGACGGGACCGCCCTTGTCGACGGAACCGCACCCC
CAGCCGTGAATTCGTATGGCATTGTCCCCGGCCAACAACAGCAATTCGTGCAG
CCCACGCCGAGCCCCAACAATGCTCTTGCACGAAGACAGATGAACAGAGCGC
TTGTGCCCACCGCTGCCAGGCCAAATTTCGATTCTGCCGCCGACCCCTGGTTCG
TTGGTGACGACAACGCCCTTCTGCAACAGCAGCAGCAACAACCAAACGGCAA
CATGACTGCCACCGATAACATCGAGGCGCTGGAGGAGATGGCTCGCAAGGCC
ATGAGAGAGGCGCAGCAGAAGAGGAAGCAAATTCCGCCCTTCGTCCAGAAGC
TCAGCAGGTCAGAGATTTCCGTGCCCCTCCCAAGCGATGCGATCAGCACGCTG
AGTGTGGGGATTGTTTACTGTGTGACGTACTGACCTTTGCGTAGCTTTTTGGACG

ACGACAAGAACTCCGACCTGATTCGATGGTCCGAGAAGGGTGATTCGTTCATC
GTTCTCGACGAGGACGAGTTCGCGAAGAAGCTCATTCCCGATCTGTTCAAGCA
CAACAACTACGCCTCATTTGTGCGACAACTCAACATGTACGGCTTCCACAAGC
GCGTTGGCTTGTCGGACAACTCCATGCGCGCGAGCGAGCGGAAGAACAAGAG
CCCCAGCGAGTACTCGAACCCGTTCTTCCGTCGCGGCCACCCGAATCTGCTGTG
GCTCATCAACAAGCCCAAGAGCGGCAACAAGGGCAAGAAAGGCGCCAAGAA
TGTAGAGATCGAGGGCGACAGCGAGGAGGATGTTGTTATCGATGACGGGGTC
ACACAAGGACTCGGCGCCACCAGCGCTCCGTCTAGCAGAGCCCTCCCGCCTGC
GGGGGACATGCCCATCCAGAAGAAGGAGATGGCCCTCATTCGCGAAGAGTTG
GCCAAGCTGCGGGATCAGCAAAGGTTGATTATGTCGGCCATCCAGCGGATCCA
GCAAGATAACTCGGCACTATACAATCAGGCCGTCGTCTTCCAGAGCCAGCACG
ATCGTCACCAAAATTCTATCAACGCCATCCTCAACTTCCTGGCCAACGTCTTTA
GGAAGACGCTCGAAGATCAGGCAGGCGCTCAAAGCGTGAACGACTTGCTGGC
GAGCATCATCCCCAATGCCAACAACAACAGCTCTGTGCCTCAGGGCAGTGTGG
TGGACCTTGGAGACTACATCCAGTCGCAGTCTGCGGCGAATAACAACATGAAC
CTCGCGAAGCGTAGACAAGCGCTTCTGCCGCCAATTCCAAACGGCCGTGCCAA
CACGGTTTCTCCATCCCCAGCATCTAGCGCGCCCACGCCCCAGCCGTACAACA
ACCGTGAGATGGGCACAGTAACAGAGCTCTTCGACACGTCACCTGGAGACCAT
GCATCCTCCCCCAGCAACTACCTGGCTCAGGAGCTTGAGACCAACCCCCACGA
GAGCATGATGAAGATCATCAACAACACCAACGCAAACCATGCAAACCAATCG
TCCAACATTGACCTGCCCGAGGTCGTGAACAACACCCCTGTCACCATGAGCAA
TGACCAGCGCACCAAGATGCTCAACATCATGGCTGGCCGTTCCGCGACGCCCA
CCAGCAACACGCCAACAACAAGGCCCTCCGTCTCCGCTTCTCCCAGACCGCCG
GCTCCATCTGTCAGCGCTACCACCGACGCGCCTTCCATGCCCGCCACATCAGG
CCTTTCACTCTCGCCTATCATGGGTTCAGCCGAACCTCCGCCTTCCCTGCAGCA
TATCCAATACAACCAAGCCGACTTGGCCGCTCTTCAGCGGATGCAAGAAGAGC
AAGCTGCCAAGCTGGACCACCTTTCAAGCATGCTGGGCCCCCTGAGCCCCTCC
GGTCGGATTCCTGGCATTGACGAGAACGGCAACATCAATCACCAGAACGGCT
ACTTTGGGTCTGACCTTGACATTGACCAGTTCCTCAACCAAGATGCTTTCCAAG
GTGATGATGCCTTCCAAGGGGGCGTCTTCCCGGGAGATGGAACCGACTTCAAC
TTTTCCCTGGACGCTGCTACACCCAACGGCGGTGTTGCGGCTGCCAATGCAACC
AACACTCCTAGTGAGAGCAGTGGAACGGAAGAAATCGCACGCGACGGCTTTG
ACCCGTACACTGGCAGTCCTGATCGTGGCACTAAAAGGCGGCGCGTCGGCTGA

>CgHSF1 Amino acid sequence
MPMPAVQQSFPSPTGDQFLRWGGADGTALVDGTAPPAVNSYGIVPGQQQQFVQP
TPSPNNALARRQMNRALVPTAARPNFDSAADPWFVGDDNALLQQQQQQPNGN
MTATDNIEALEEMARKAMREAQQKRKQIPPFVQKLSSFLDDDKNSDLIRWSEKGDS
FIVLDEDEFAKKLIPDLFKHNNYASFVRQLNMYGFHKRVGLSDNSMRASERKNKSP
SEYSNPFFRRGHPNLLWLINKPKSGNKGKKGAKNVEIEGDSEEDVVIDDGVTQGLG
ATSAPSSRALPPAGDMPIQKKEMALIREELAKLRDQQRLIMSAIQRIQQDNSALYNQ
AVVFQSQHDRHQNSINAILNFLANVFRKTLEDQAGAQSVNDLLASIIPNANNNSSV
PQGSVVDLGDYIQSQSAANNNMNLAKRRQALLPPIPNGRANTVSPSPASSAPTPQP
YNNREMGTVTELFDTSPGDHASSPSNYLAQELETNPHESMMKIINNTNANHANQS
SNIDLPEVVNNTPVTMSNDQRTKMLNIMAGRSATPTSNTPTTRPSVSASPRPPAPSV
SATTDAPSMPATSGLSLSPIMGSAEPPPSLQHIQYNQADLAALQRMQEEQAAKLDH
LSSMLGPLSPSGRIPGIDENGNINHQNGYFGSDLDIDQFLNQDAFQGDDAFQGGVF
PGDGTDFNFSLDAATPNGGVAAANATNTPSESSGTEEIARDGFDPYTGSPDRGTKR
RRVG
